# Global public health challenges require stronger European collaboration

**DOI:** 10.1093/eurpub/ckab149

**Published:** 2022-02-01

**Authors:** Rok Hrzic, Timo Clemens, Helmut Brand

**Affiliations:** 1 Department of International Health, Care and Public Health Research Institute – CAPHRI, Maastricht University, Maastricht, The Netherlands; 2 Prasanna School of Public Health, Manipal Academy of Higher Education, Manipal, India

Ophoven is a village on the border of Germany and the Netherlands, near the river Roer. It was inundated by the floods in Western Europe on July 13–16 that left almost 200 dead. The proximal cause of the disaster in Ophoven was the failure of the Roer dam. According to the mayor of a nearby town, this failure was in part caused by the Dutch town of Venlo, where the Roer joins with another river, engaging the locks on their own dam. Whether or not this narrative is accurate—the mayor reversed his position soon after—there is a sense of failure to effectively coordinate across national borders.

The extreme rainfall leading to the floods is widely seen as climate change making itself felt in the everyday life of the world’s richest countries. More frequent experiences of extreme weather and the past 18 months of the COVID-19 pandemic are leading to an overdue realization that no place on Earth can escape the consequences of planet-wide challenges. This may have accelerated the pace and scope of the policy response. On July 14, the European Commission announced further details on the European Green Deal in a communication entitled ‘Fit for 55’, proposing a set of climate, energy, transport and taxation policies that aim to reduce greenhouse emissions by 55% by 2030.^1^ While economic incentives are a part of the puzzle, they alone will not suffice. Better governance structures are required as well.

Climate change, emerging pandemics and antimicrobial resistance are planet-wide challenges that care little for national borders. The continued failure to effectively tackle them betrays the limits of a nation state centred global governance framework. Part of the European Union response is a proposal for a new regulation on serious cross-border threats to health, which would strengthen existing EU agencies, data sharing between Member States and give the EU the power to declare emergencies.^2^ However, we would argue that we also need more cross-border cooperation between regional and local authorities independent from national governments or supranational institutions.

Examples of effective regional cross-border collaboration already exist. The Meuse–Rhine Euroregion, the cross-border area of Belgium, the Netherlands and Germany, has a history of cooperation in disaster preparedness. EMRIC, the cross-border centre for incident control and crisis management, brings together first responders in the cross-border region.^3^ These lines of communication and cooperation between regional authorities may well have saved lives during the latest disaster. More should be done to strengthen initiatives like EMRIC, and we should consider founding new ones that specialize in climate adaptation and pandemic preparedness. However, a key lesson from the recent floods is that cross-border initiatives also need to be more effectively integrated into decision-making at the regional level and that the decision-making process itself needs to be made more transparent.

The European Flood Awareness System (EFAS) is a Europe-wide collaboration built in response to the 2002 flooding of the Danube and Elbe rivers. EFAS’ foresight is strengthened by the fact that the collaboration has access to better data and modelling capacities than any individual government.^4^ As early as July 10, it issued several flood warnings, which did not elicit an adequate response by the national and regional authorities. Why this occurred is unclear for now. One contributing factor may be mobile number-based public warning systems with insufficient reach. Cell broadcasts are regulated by Article 110 of the European Electronic Communications Code,^5^ for which the German government chose for an exemption provision and relies instead on a mobile application system (NINA) that only 10 million users (about 12% of the German population) have downloaded to date. What the situation does suggest is that the status quo is insufficient and that communication between cross-border initiatives and national and regional decision-makers needs to be strengthened and the decision-making process made more transparent to the public.

We must learn from experience if we are to survive an increasingly complex world faced with threats of planetary scale. One key lesson we draw is that the nation state centred international governance framework is not sufficient to tackle climate change, future pandemics and other global challenges that care little for national borders. We propose to strengthen cross-border collaboration between regional authorities by empowering existing and forming new cross-border initiatives and call for them to become an integral part of regional decision-making that is made more transparent to the public.

*Conflicts of interest*: None declared.
